# Photokératoconjonctivite par coup d′arc

**DOI:** 10.11604/pamj.2020.36.42.19549

**Published:** 2020-05-27

**Authors:** Brahim Salem Joumany, Sidi Dahi, Mahdi Khamaily, Iman Tarib, Nisrine Laaribi, Karim Reda, Abdelbarre Oubaaz

**Affiliations:** 1 Service d'Ophtalmologie, Hôpital Militaire d′Instruction Mohammed V, Faculté de Médecine et de Pharmacie, Université Mohammed V, Rabat, Maroc

**Keywords:** Photokératoconjonctivite, coup d′arc, photokératite, Keratoconjunctivitis photoelectrica, arc eye, photokeratitis

## Abstract

La photokératite est une kératite douloureuse causée par l'exposition non-protégée des yeux aux rayons ultraviolets (UV). On parle de «coup d'arc» quand la photokératite est induite par le rayonnement UV émis par l′arc électrique pendant le soudage à l′arc électrique. Nous rapportons le cas d′un patient de 35 ans, sans antécédents notables, cavalier de profession qui se dit avoir regardé pendant quelques minutes, un arc électrique durant le soudage des portes du stable suite à laquelle, il a présenté une douleur oculaire bilatérale à type de brûlure associé à des larmoiements photophobie et blépharospasme. Son examen clinique a montré une acuité visuelle avec correction à 8/10 et 9/10, une hyperhémie conjonctivale avec une kératite érosive ponctuée limité à la fente palpébrale après instillation de la fluorescéine. Vu le contexte, le diagnostic d'une kératoconjonctivite par coup d'arc a été retenu. Le patient a été mis sous antibiotique topique, agents mouillants et cicatrisants. L′évolution a été marquée, par la disparition totale des signes avec une acuité visuelle qui est remonté à 10/10 en bilatérale. A travers ce cas, on illustre l′intérêt d′une prévention par un port d′équipements de protection adaptés.

## Abstract (en)

Photokeratitis is a painful keratitis caused by exposure of insufficiently protected eyes to the ultraviolet (UV) rays. We talk about “arc eye” when photokeratitis is caused by UV rays emitted by electric arc during electric arc welding process. We here report the case of a 35-year old rider, with no previous medical-surgical history, who had looked at an electric arc for a few minutes while the doors of his building were welded. He had bilateral burning in his eyes associated with lacrimation, photophobia and blepharospasm. Clinical examination showed corrected visual acuity of 8/10 and 9/10, conjunctival hyperemia with punctate erosive keratitis limited to palpebral fissure after the use of fluorescein. Given patient's clinical picture, keratoconjunctivitis photoelectrica (arc eye) was diagnosed. Treatment was based on topical antibiotics, wetting agents as well as agents with healing properties. Outcome was marked by total disappearance of the signs with restoration of bilateral visual acuity (10/10). This study highlights the role of prevention using appropriate protection equipment.

## Introduction

La photokératite est une kératite douloureuse causée par l'exposition non-protégée des yeux aux rayons ultraviolets (UV). On parle de « coup d'arc » quand la photokératite est induite par le rayonnement UV émis par l'arc électrique pendant le soudage à l'arc électrique. Nous rapportons le cas d'un patient qui présente un coup d'arc.Patient et observation

**Figure 1:**
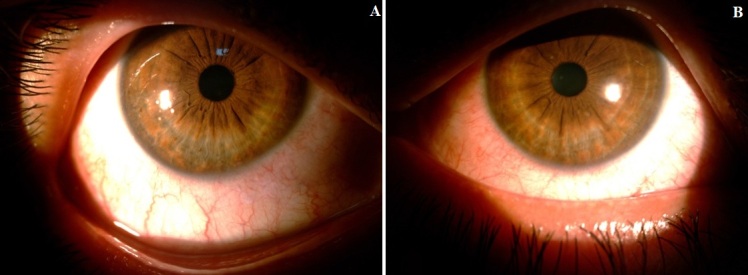
Hyperhémie conjonctivale au niveau de l’oeil droit (A) et gauche (B); il est à noter que le blépharospasme gêne l’examen

**Figure 2:**
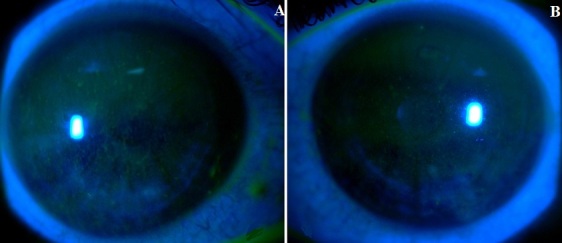
Images montrant une kératite ponctuée érosive bilatérale après instillation de la fluorescéine: A) œil droit; B) œil gauche

Patient âgé de 35 ans, sans antécédents notables, cavalier de profession qui se dit avoir regardé pendant quelques minutes, un arc électrique durant le soudage des portes du stable. Dix heures plus tard, une douleur oculaire bilatérale à type de brûlure, réveille le patient au milieu de la nuit, motivant sa consultation aux urgences. Il se plaint de larmoiement, de photophobie et de blépharospasme. La meilleure acuité visuelle avec correction correspond à 8/10 et à 9/10, au niveau de l'œil droit et gauche respectivement. L'examen à la lampe à fente révèle une hyperhémie conjonctivale, une discrète diminution du reflet cornéen (Figure 1) avec un aspect de kératite érosive ponctuée limité à la fente palpébrale après instillation de la fluorescéine (Figure 2). Le reste de l'examen est sans particularités. Le diagnostic d'une kératoconjonctivite par coup d'arc est retenu. Le patient est mis sous antibiotique en topique, agents mouillants et cicatrisants avec un repos dans une chambre sombre. L'évolution est marquée après 72 heures, par la disparition totale des signes fonctionnelles et des lésions visibles initialement à l'examen, l'acuité visuelle est remontée à 10/10 en bilatéral.

## Discussion

Le coup d'arc des soudeurs entraine une kératite ponctuée érosive après quelques heures de latence; typiquement: la douleur violente réveille le patient à 3 h du matin. Le patient se plaint généralement d'un œil rouge douloureux avec un larmoiement et une photophobie intense. La photokératite correspond à une réponse cornéenne aux rayonnements ultraviolets intenses. Le principal site de dégâts est l'épithélium, cependant, des études suggèrent le rôle de l'apoptose des kératocytes des cellules endothéliales [1]. La gravité des dommages histologiques est en corrélation avec l'intensité du rayonnement UV et la durée d'exposition [2]. Fort heureusement, les signes sont réversibles en 48 à 72 heures. Il faut encore une fois souligner l'intérêt de la prévention.

## Conclusion

Le coup d'arc est une pathologie souvent bénigne où la cicatrisation cornéenne se fait en 48h. Ce tableau, si bruyant soit tel, peut être prévenu par un simple port d'équipements de protection adaptés.

## Conflits d’intérêts

Les auteurs ne déclarent aucun conflit d'intérêts.
